# 1885

**Published:** 1885-01-20

**Authors:** 


### 1885.

With this number commences the fifteenth volume of our journal. We extend to our numerous readers and advertisers the "complements of the season," and return our thanks for their continued confidence and patronage.

Our journal is flourishing, and yet there are a great many physicians in our land who do not take it; and it is a shame to have to say that many practitioners do not take any journal, and do not read at all. Of course, they cannot know what advances are being made, and are unable to give their patients the benefits of the improvements that are constantly taking place. Is this right? Indeed, is it not manifestly wrong to set yourself up as a practicing physician and yet fail to avail yourself of a knowledge and use of all the best and latest applianees in the medical art? It requires a nice discrimination to detect the difference, morally, between letting a patient die from the lack of attainable information, and destroying him by improper medication.

The local physician seeks to avail himself of all the known methods for the relief of his patient. This he cannot do without taking one or more good medical journals and keeping himself well posted in the progress of Medical Science.

